# Aminoglycoside therapy for childhood urinary tract infection due to extended-spectrum β-lactamase-producing *Escherichia coli* or *Klebsiella pneumoniae*

**DOI:** 10.1186/s12879-015-1153-z

**Published:** 2015-10-13

**Authors:** Seung Beom Han, Sung Chul Lee, Soo Young Lee, Dae Chul Jeong, Jin Han Kang

**Affiliations:** Department of Pediatrics, College of Medicine, The Catholic University of Korea, Seoul, Republic of Korea; The Vaccine Bio Research Institute, College of Medicine, The Catholic University of Korea, Seoul, Republic of Korea; Department of Pediatrics, College of Medicine, Incheon St. Mary’s Hospital, The Catholic University of Korea, Dongsu-ro 56, Bupyeong-gu, Incheon, 403-720 Republic of Korea

**Keywords:** Urinary tract infection, *Escherichia coli*, *Klebsiella pneumoniae*, Beta-lactamase, Child

## Abstract

**Background:**

The rate of urinary tract infections (UTIs) due to extended-spectrum β-lactamase (ESBL)-producing bacterial strains requiring carbapenem therapy has been increasing in children. This study was conducted to evaluate the effect of non-carbapenem antibiotic therapy on childhood UTIs caused by ESBL-producing *Escherichia coli* or *Klebsiella pneumoniae.*

**Methods:**

Medical records of children diagnosed with febrile UTIs due to *E. coli* or *K. pneumoniae* between 2010 and 2014 were retrospectively reviewed. The enrolled children were divided into two groups: the ESBL group and the non-ESBL group. Clinical characteristics and therapeutic responses were compared between the two groups.

**Results:**

A total of 211 episodes of UTI (204 caused by *E. coli*; seven caused by *K. pneumoniae*) were identified in 205 children. Twenty-two (10.4 %) episodes were categorized into the ESBL group. There was no significant difference in the type of antibiotic administered between the two groups. No carbapenems were administered; however, aminoglycosides were administered for 79.1 % of the total episodes. Although empirical antibiotics were appropriate for more episodes in the non-ESBL group compared with the ESBL group (100.0 % vs. 90.9 %, *p* = 0.011), there were no significant differences in the frequency of defervescence, bacterial eradication from the urine, acute pyelonephritis and vesicoureteral reflux or fever duration between the two groups.

**Conclusions:**

Non-carbapenem antibiotics showed favourable therapeutic effects on childhood UTIs caused by ESBL-producing strains. Aminoglycosides can be an alternative to carbapenems in such cases.

## Background

Urinary tract infections (UTIs) are one of the most common bacterial infections in children, and are most frequently caused by *Enterobacteriaceae*, including *Escherichia coli* and *Klebsiella pneumoniae* [1, 2]. Third-generation cephalosporins have been widely used for the treatment of community-acquired UTIs; however, the rate of UTIs caused by extended-spectrum β-lactamase (ESBL)-producing *E. coli* or *K. pneumoniae*, which are resistant to cephalosporins, has been increasing in adults as well as children [3–6]. In addition, UTIs due to ESBL-producing strains may cause prolonged hospital stay and an increase in medical costs [2, 5, 6]. Although carbapenems are recommended for treating infections caused by ESBL-producing strains [7, 8], their use should be restricted given the concerns regarding increasing resistance [9, 10]. Fortunately, non-carbapenem antibiotic therapy has shown favourable therapeutic effects on UTIs due to ESBL-producing strains in adults; however, studies in children are scarce [11, 12].

This retrospective study compared clinical and microbiological responses to non-carbapenem antibiotic therapy between febrile UTIs caused by ESBL-producing *E. coli* or *K. pneumoniae* and febrile UTIs caused by non-ESBL-producing strains in healthy children. In addition, the efficacy of non-carbapenem antibiotics administered to children with UTIs caused by ESBL-producing strains was also evaluated.

## Methods

### Patients and study design

Medical records for children (<18 years of age) diagnosed with a febrile UTI caused by *E. coli* or *K. pneumoniae* in the inpatient and outpatient clinics of the Department of Pediatrics at Seoul St. Mary’s Hospital, Seoul, Republic of Korea, between January 2010 and December 2014 were retrospectively reviewed. To avoid the inclusion of hospital-acquired UTIs, data were excluded if urine cultures were performed 48 h or more after admission to hospital. Data for immunocompromised children with underlying haematological/oncological diseases as well as data for children with underlying chronic neurological disorders, haemodynamically unstable congenital heart diseases, underlying functional and anatomical urological disorders, indwelling urinary catheters, or other febrile illnesses with accompanying pyuria (e.g. polymerase chain reaction-confirmed respiratory viral infection or Kawasaki disease) were also excluded. A UTI was diagnosed in febrile children when the following criteria were met: 1) positive results on a urine dipstick test (leukocyte esterase or nitrite) or a white blood cell count > 10/high power field on urinary flow cytometry analysis, 2) identification of a single pathogen with > 100,000 colony forming units/mL in a urine culture. Urine samples were collected from non-toilet-trained children by applying a sterile urine bag over the perineum after skin disinfection, and clean, voided midstream urine was collected from toilet-trained children. The data for the enrolled children were divided into ESBL and non-ESBL groups based on the ESBL positivity of the identified urinary pathogen. The clinical characteristics of the two groups were then compared. This study was approved by the Institutional Review Board of Seoul St. Mary’s Hospital (Approval No.: KC15RISI0119). Informed consent was waived for this study.

### Comparison of demographic and clinical characteristics

Age, sex, identified urinary pathogens, previous history of UTIs, and fever duration (before and after antibiotic therapy) were compared between the two groups. Acute pyelonephritis (APN) was diagnosed according to ^99m^technetium dimercaptosuccinic acid (DMSA) scan results. Vesicoureteral reflux (VUR) was diagnosed according to voiding cystourethrography (VCUG) results. The rates of APN and VUR were compared between the two groups. Almost all pregnant women in the Republic of Korea receive regular antepartum care and repeated foetal ultrasonography, and therefore, clinicians in our hospital focus on identifying APN rather than urogenital structural anomalies in children diagnosed with UTIs. DMSA scans, which are more sensitive in diagnosing APN than renal ultrasonography, are performed for most UTI episodes in our hospital for such a purpose.

### Comparison of administered antibiotic therapy

Antibiotics that were initially administered to febrile children before the identification of the urinary pathogen and its antibiotic susceptibility were defined as empirical antibiotics. To evaluate the therapeutic effect of the empirical antibiotics, the type and appropriateness of the antibiotics, duration of antibiotic therapy, and frequency of defervescence and bacterial eradication from the urine during empirical therapy were compared between the two groups. Empirical antibiotic therapy was considered appropriate if the identified uropathogen was susceptible to at least one of the administered empirical antibiotics. If the identified pathogen was non-susceptible to all of the empirical antibiotics, the therapy was considered inappropriate.

### Comparison of antibiotic susceptibility rates

Urine cultures and the identification of urinary pathogens were performed using the VITEK®2 automated system (bioMérieux, Hazelwood, MO, USA). Antibiotic susceptibility tests and the identification of ESBL were performed using antibiotic susceptibility test cards (VITEK®2 automated system). In cases for which a cefotaxime susceptibility test was not performed, ceftazidime susceptibility results were applied to determine cefotaxime susceptibility, and cefotaxime or ceftazidime susceptibility results were applied to determine susceptibilities to oral third-generation cephalosporins (e.g. cefpodoxime, cefditoren, and cefixime). Susceptibility to amoxicillin/clavulanate and susceptibility to ampicillin/sulbactam were considered identical. Susceptibility rates were compared between the two groups.

### Statistical analysis

A *χ*^2^ test was used to compare categorical variables while numerical variables were compared using a Mann–Whitney test. Statistical analyses were performed using SPSS version 15.0 (SPSS Inc., Chicago, IL, USA) and a two tailed *p* < 0.05 was considered a significant result.

## Results

During the study period, 211 episodes of febrile UTI caused by *E. coli* or *K. pneumoniae* were diagnosed in 205 children. Eleven (5.2 %) of these episodes were treated solely at the outpatient clinic. The median age of the enrolled children was 5.0 months (interquartile range, IQR: 3.0–7.0), and 128 (60.7 %) cases were boys. Of the 211 episodes, 204 (96.7 %) were caused by *E. coli* while seven (3.3 %) were caused by *K. pneumoniae* (Table 1). Twenty-two episodes were caused by ESBL-producing strains, and 21 (95.5 %) of these were caused by ESBL-producing *E. coli* (Table 1). Bacteraemia accompanied eight (3.8 %) of these episodes (seven in the non-ESBL group and one in the ESBL group), although none needed intensive care.

**Table 1:** Characteristics of children with urinary tract infection caused by *E. coli* or *K. pneumoniae*

Factor	Non-ESBL group	ESBL group	*p-*value
	(*n* = 189)	(*n* = 22)	
Sex (male)	114 (60.3)	14 (63.6)	0.763
Age (months)	4.0 (3.0–7.0)	5.0 (4.0–6.5)	0.203
Pathogen			0.543
*E. coli*	183 (96.8)	21 (95.5)	
*K. pneumoniae*	6 (3.2)	1 (4.5)	
Methods of urine collection			0.119
Urine bag	174 (92.1)	18 (81.8)	
Mid-stream urine	15 (7.9)	4 (18.2)	
Previous history of urinary tract infection	9 (4.8)	1 (4.5)	1.000
Fever duration at the beginning of antibiotic therapy (days)^a^	2.0 (1.0-3.0)	2.0 (1.0-3.0)	0.923
Acute pyelonephritis on DMSA scan^b^	127 (80.4)	17 (81.0)	1.000
Vesicoureteral reflux on VCUG^c^	18 (15.9)	4 (23.5)	0.488

Data are median (interquartile range) or no. (%) of cases

*ESBL* extended-spectrum β-lactamase; *DMSA* ^99m^technetium dimercaptosuccinic acid; *VCUG* voiding cystoutrthrography

^a^The accurate duration of fever at the beginning of antibiotic therapy was not determined in one child in the non-ESBL group, who was treated in the outpatient clinic

^b^DMSA scan was performed in 179 children (158 in the non-ESBL group, 21 in the ESBL group)

^c^VCUG was performed in 130 children (113 in the non-ESBL group, 17 in the ESBL group)

### Comparison of demographic and clinical characteristics

Age, sex, and causative pathogens were compared between the ESBL group (22 episodes, 10.4 %) and the non-ESBL group (189 episodes, 89.6 %), and there were no significant differences between the two groups (Table 1). A previous history of UTIs was identified for 10 (4.7 %) of the episodes; however, the frequency of recurrence was not significantly different between the two groups. The median duration of fever at the beginning of antibiotic therapy was 2.0 days (IQR: 1.0-3.0) and was not significantly different between the two groups (Table 1). APN was diagnosed in 144 (80.4 %) of the 179 episodes for which a DMSA scan was performed and VUR was diagnosed in 22 (16.9 %) of the 130 episodes for which VCUG was performed. The APN and VUR frequencies were not significantly different between the two groups (Table 1).

### Comparison of clinical and microbiological responses to antibiotic therapy

Cephalosporins or β-lactam/β-lactamase inhibitor combinations (BL/BLIs) were empirically administered in addition to aminoglycosides in 167 (79.1 %) of the UTI episodes. The type of empirical antibiotics was not significantly different between the two groups (Table 2). The empirical aminoglycosides were administered for a median of 6.0 days (IQR: 4.5–7.0). The administration of empirical aminoglycosides was discontinued during hospitalisation for 16 (9.6 %) episodes based on the antibiotic susceptibility results, and it was continued during the entire period of hospitalisation in the remaining episodes. Serum creatinine levels were re-checked during aminoglycoside therapy in 81 episodes (72 in the non-ESBL group, nine in the ESBL group), but, none showed a rising serum creatinine level that was more than twice that of baseline. Empirical antibiotics were appropriate in all episodes for the non-ESBL group and 90.9 % for the ESBL group (*p* = 0.011, Table 2). Fever disappeared in 92.3 % of UTI episodes during empirical antibiotic therapy. There were no significant differences between the two groups in frequency of defervescence or duration of fever during empirical antibiotic therapy (Table 2). Urine cultures were repeated after empirical antibiotic therapy (median 2 days, IQR: 2–3) for all but 14 episodes (13 in the non-ESBL group and one in the ESBL group). Urinary pathogens were eradicated for 99.0 % of the episodes for which repeated urine cultures were performed. The rate of bacterial eradication during empirical antibiotic therapy was not significantly different between the two groups (Table 2). Among the 200 hospitalised cases, return visits to the outpatient clinic were made for 193 (96.5 %) of the episodes within a median of 7.0 days (IQR: 5.0–8.0) after discharge from hospital, and there were no re-emerging fever or urological symptoms during the period.

**Table 2:** Empirical antibiotic therapy and therapeutic responses in children with urinary tract infection caused by *E. coli* or *K. pneumoniae*

Factor	Non-ESBL group	ESBL group	*p-*value
	(*n* = 189)	(*n* = 22)	
Administered empirical antibiotics			0.814
With aminoglycoside			
Third-generation cephalosporin + aminoglycoside	111 (58.7)	13 (59.1)	
Aminopenicillin/β-lactamase inhibitor + aminoglycoside	34 (18.0)	4 (18.2)	
Second-generation cephalosporin + aminoglycoside	4 (2.1)	1 (4.5)	
Without aminoglycoside			
Third-generation cephalosporin	25 (13.2)	3 (13.6)	
Aminopenicillin/β-lactamase inhibitor	2 (1.1)	1 (4.5)	
Second-generation cephalosporin	1 (0.5)	0 (0.0)	
Others	10 (5.3)	0 (0.0)	
No therapy	2 (1.1)	0 (0.0)	
Appropriateness of empirical antibiotic therapy^a^	182 (100.0)	20 (90.9)	0.011
Antibiotic duration (days)			
Total	13.0 (12.0–14.0)	14.0 (13.0–16.0)	0.048
Intravenous^b^	6.0 (5.0–7.0)	6.0 (5.0–7.0)	0.742
Oral^c^	7.0 (7.0–9.0)	7.0 (6.0–10.5)	0.477
Aminoglycosides^d^	6.0 (4.0–7.0)	6.0 (5.0–7.0)	0.170
Fever duration after antibiotic therapy (days)^e^	1.0 (0.0–2.0)	1.0 (0.0–1.3)	0.573
Response to empirical antibiotic therapy			
Disappearance of fever^e^	172 (92.5)	20 (90.9)	0.680
Bacterial eradication on the urine^f^	174 (98.9)	21 (100.0)	1.000
Follow-up time of urine culture (days)^f^	2.0 (2.0–3.0)	2.0 (1.0–2.5)	0.611

Data are median (interquartile range) or no. (%) of cases

*ESBL* extended-spectrum β-lactamase

^a^The appropriateness of empirical antibiotic therapy was determined in 204 children (182 in the non-ESBL group, 22 in the ESBL group)

^b^Eleven children of the non-ESBL group, who did not received antibiotic therapy or received exclusively oral antibiotic therapy, were excluded

^c^Six children, who received exclusively intravenous antibiotic therapy, were excluded (Five in the non-ESBL group, One in the ESBL group)

^d^The duration of aminoglycoside therapy was determined in 167 children (149 in the non-ESBL group, 18 in the ESBL group)

^e^The accurate time of defervescence was not determined in three children in the non-ESBL group, who was lost to follow-up in the outpatient clinic

^f^Microbiological response to first line antibiotics was determined in 197 children (176 in the non-ESBL group and 21 in the ESBL group) in whom repeated urine cultures were performed before antibiotic change

All episodes in the ESBL group were initially treated with intravenous antibiotics, and oral antibiotics were administered after discharge from hospital for 21 (95.5 %) episodes. Cefotaxime and amikacin were administered for 13 (59.1 %) episodes, amoxicillin/clavulanate and amikacin for four (18.2 %), cefotaxime for three (13.6 %), cefuroxime and amikacin for one (4.5 %), and amoxicillin/clavulanate for one (4.5 %). However, none of the ESBL group received carbapenem therapy. Cefuroxime susceptibility testing was not performed in the one episode for which cefuroxime was empirically administered. Empirically administered amoxicillin/clavulanate and cefotaxime were non-susceptible to identified pathogens in 16 (76.2 %) of the remaining 21 episodes. Nevertheless, the non-susceptible amoxicillin/clavulanate and cefotaxime had been administered until discharge from hospital in all of the 16 episodes based on favourable clinical responses. Amikacin was susceptible to identified pathogens in all of the 18 episodes in which it was empirically administered. Empirical cefotaxime administered without aminoglycosides was inappropriate in two (66.7 %) of three episodes; however, fever disappeared 2 days after cefotaxime therapy, and second urine cultures became sterile in both cases. Cefixime was administered for nine (42.9 %) of the 21 episodes for which oral antibiotics were administered after discharge from hospital, amoxicillin/clavulanate for five (23.8 %), trimethoprim/sulfamethoxazole for four (19.0 %), and cefditoren, cefpodoxime, and ciprofloxacin for one (4.8 %) episode each. Although the identified uropathogens were not susceptible to administered oral antibiotics in 10 of the 11 episodes for which oral third-generation cephalosporin was administered and in two of the five episodes for which oral amoxicillin/clavulanate was administered, no re-emerging fever or urological symptoms were observed during oral antibiotic therapy.

### Comparison of antibiotic susceptibility rates

The rates of susceptibility to empirically administered BLs and BL/BLIs were significantly different between the non-ESBL group (95.9 %, 162/169) and ESBL group (23.8 %, 5/21, *p* < 0.001). However, 99.3 % (148/154) of the episodes in the non-ESBL group and 100.0 % (18/18) in the ESBL group were susceptible to empirically administered aminoglycosides (*p* = 1.000).

Antibiotic susceptibility rates tended to be lower in the ESBL group compared with the non-ESBL group (Table 3). However, susceptibility rates to BL/BLIs (e.g. amoxicillin/clavulanate and piperacillin/tazobactam) and to aminoglycosides (e.g. gentamicin, tobramycin, and amikacin) were not significantly different between the two groups (Table 3). No carbapenem-non-susceptible isolates were identified during the study period.

**Table 3:** Comparison of antibiotic susceptibility rates between the non-ESBL and ESBL groups

Antibiotics	Non-ESBL group	ESBL group	*p-*value
	(*n* = 189)	(*n* = 22)	
Penicillins			
Ampicillin	44/131 (33.6)	0/18 (0.0)	0.003
Piperacillin	40/103 (38.8)	0/11 (0.0)	0.008
Cephalosporins			
Cefoxitin	127/130 (97.7)	16/18 (88.9)	0.112
Cefotaxime	142/144 (98.6)	2/19 (10.5)	<0.001
Caftazidime	185/189 (97.9)	10/22 (45.5)	<0.001
Cefepime	186/189 (98.4)	10/22 (45.5)	<0.001
β-lactam/β-latamase inhibitor combinations			
Amoxicillin/clavulanate	107/131 (81.7)	12/18 (66.7)	0.205
Piperacillin/tazobactam	177/187 (94.7)	18/21 (85.7)	0.131
Carbapenems			
Imipenem	189/189 (100.0)	22/22 (100.0)	NA
Meropenem	189/189 (100.0)	22/22 (100.0)	NA
Aminoglycosides			
Gentamicin	158/188 (84.0)	16/22 (72.7)	0.228
Tobramycin	87/102 (85.3)	9/11 (81.8)	0.670
Amikacin	188/189 (99.5)	22/22 (100.0)	1.000
Isepamicin	58/58 (100.0)	4/4 (100.0)	NA
Fluoroquinolones			
Ciprofloxacin	128/145 (88.3)	8/15 (53.3)	0.002
Levofloxacin	42/44 (95.5)	5/7 (71.4)	0.086
Others			
Trimethoprim/sulfamethoxazole	137/188 (72.9)	13/22 (59.1)	0.176
Colistin	58/58 (100.0)	4/4 (100.0)	NA
Minocycline	47/58 (81.0)	4/4 (100.0)	1.000

Data are no. (%) of cases

*ESBL* extended-spectrum β-lactamase; *NA* not available

## Discussion

In the present study, therapeutic responses of febrile UTIs to antibiotic therapy were compared between UTIs caused by ESBL-producing strains and non-ESBL-producing strains. Prescribing carbapenems has been restricted and allowed only with the permission of the infectious disease (ID) physicians in our hospital. Because attending physicians discussed with ID physicians whether to administer carbapenems or not to their patients, and ID physicians decide based on the patient’s response to empirically administered antibiotics, no children received carbapenems in our study. There were no significant differences in the clinical and microbiological responses of febrile UTIs to non-carbapenem antibiotic therapy between the ESBL and non-ESBL groups. There was also no significant difference in the frequency of APN or VUR between the two groups.

ESBLs are resistant to aminopenicillins and ureidopenicillins as well as extended-spectrum β-lactam agents; ESBLs also usually present resistance to other classes of antibiotics, such as quinolones and trimethoprim/sulfamethoxazole [8, 13, 14]. Therefore, the broadest-spectrum antibiotic agents, carbapenems, are recommended to treat infections caused by ESBL-producing bacteria [7, 8]. Risk factors for UTIs due to ESBL-producing strains in children, including being less than 1 year old, having a recent history of hospitalisation and antibiotic therapy with BLs, the presence of underlying disease, and *K. pneumoniae* infection, have been reported [1, 4–6, 15, 16]. Given that carbapenem use should be restricted and most cases of UTIs are not life-threatening, previous studies have focused on non-carbapenem antibiotics in UTIs due to ESBL-producing strains [11, 12, 17–19]. However, few studies have examined the therapeutic efficacy of non-carbapenem antibiotics in childhood UTIs caused by ESBL-producing strains [11, 12]. Cephamycins, which are stable against ESBL, as well as fosfomycin and nitrofurantoin showed therapeutic effects for UTIs caused by ESBL-producing strains [18, 20, 21]. However, data concerning their efficacy and safety in children were insufficient for these antibiotics. In addition, cephamycin therapy tends to provoke inducible resistance to itself and occasionally to carbapenems; therefore, cephamycin monotherapy for infections due to ESBL-producing strains has not been accepted [7, 22].

In the present study, clinical and microbiological responses to non-carbapenem antibiotic therapy were not significantly different between the ESBL and non-ESBL groups, even though inappropriate empirical antibiotics were administered in significantly more episodes in the ESBL group than in the non-ESBL group. Previous studies reported favourable outcomes of cephalosporin therapy in UTIs caused by ESBL-producing strains despite the weakness of cephalosporins against ESBL. This phenomenon may have been due to higher antibiotic concentrations in urine compared with serum [1, 12, 19]. Cefotaxime monotherapy in the ESBL group helped achieve defervescence and urinary sterilisation in the present study, but there were only two such cases. Although 50.0 % of the ESBL group received third-generation oral cephalosporins after discharge from hospital and there was no re-emergence of fever or urological symptoms during oral antibiotic therapy, most children of the ESBL group had received a median of 6.0 days of aminoglycoside therapy during their hospitalisation. Prolonged antibiotic effect of aminoglycosides should be considered. In addition, no controlled studies on the clinical efficacy of third-generation cephalosporins in UTIs caused by ESBL-producing strains have been conducted and the use of third-generation cephalosporins is significantly associated with the development of ESBL-producing strains [13, 16, 23]. Furthermore, a recent study reported that ceftriaxone therapy in murine pyelonephritis caused by ESBL-producing *E. coli* reduced the renal bacterial burden but did not alter renal inflammation [24]. This suggests that *in vivo* clinical efficacy and long-term outcomes of third-generation cephalosporin therapy in UTIs due to ESBL-producing strains should be further evaluated.

Because clavulanate, a BLI, can inhibit ESBL activity, BL/BLIs were considered an alternative to carbapenems in treating infections due to ESBL-producing strains [25, 26]. In the present study, the susceptibility rates to amoxicillin/clavulanate and piperacillin/tazobactam in the ESBL group (66.7 % and 85.7 %, respectively) were higher than those to cefotaxime, ceftazidime, and cefepime. In the present study, aminoglycosides were empirically administered for 79.1 % of the total episodes and 81.8 % of episodes in the ESBL group. Only 23.8 % of the ESBL-producing isolates in the present study were susceptible to empirically administered BLs and BL/BLIs; however, all of the ESBL-producing isolates were susceptible to empirically administered aminoglycosides. Therefore, empirically administered aminoglycosides in the ESBL group may have contributed to the non-significant findings in the clinical and microbiological responses between the ESBL and non-ESBL groups. Park et al. reported on the clinical and microbiological effects of aminoglycosides in adults and children with UTIs caused by ESBL-producing strains [17], and the efficacy of aminoglycoside combination therapy has also been demonstrated in bacteraemia caused by ESBL-producing strains [27]. In the present study, tobramycin and gentamicin, which have been used for several decades, were susceptible against > 70 % of the ESBL-producing isolates, and amikacin and isepamicin were susceptible against all of the identified ESBL-producing isolates. Therefore, empirical antibiotics for children presumably having UTIs should include aminoglycosides considering that UTIs caused by ESBL-producing strains have been increasing. Considering the possibility of having reactive pyuria due to a bacterial infection other than a UTI and urosepsis in febrile children with abnormal urinalysis and urinary microscopic examination results, empirical administration of BLs or BL/BLIs in combination with aminoglycosides and adjustment of antibiotics based on the results of urine culture and antibiotic susceptibility of the identified pathogens seem to be more reasonable than empirical aminoglycoside monotherapy.

Given the retrospective nature of the present study, the long-term outcomes of UTIs caused by ESBL-producing strains could not be determined. Because we could not control the administered antibiotics and none received carbapenem therapy, treatment outcomes between carbapenems and non-carbapenems could not be compared. Risk factors for UTIs due to ESBL-producing strains could not be appropriately evaluated in the present study because of the small number of cases in the ESBL group. Although therapeutic effect of non-carbapenems in UTIs due to ESBL-producing strains may change as the number of these cases is increasing, other recent studies also showed a favourable therapeutic effect of non-carbapenems in UTIs caused by ESBL-producing strains [11, 12, 17–21]. However, further studies including a large number of cases should be necessary to exactly define the effectiveness of non-carbapenems in UTIs due to ESBL-producing strains. The frequencies of APN and VUR may be inaccurate because the DMSA scan and VCUG were not performed universally, but in accordance with the attending physicians’ decisions. In addition, UTIs may be over-diagnosed because most urine samples in the present study were collected using a urine bag. However, the present study examined the data from febrile children with abnormal results on urine dipstick tests or flow cytometric analyses in addition to positive urine cultures. APN based on the results of a DMSA scan was diagnosed in 80.4 % of the total 211 episodes. This rate is higher than rates reported in previous studies [2, 12]; therefore, the number of over-diagnosed UTI episodes in the present study might be small. Furthermore, although childhood UTIs need antibiotic therapy for 7 to 14 days [28], aminoglycosides can cause treatment duration-related nephrotoxicity and ototoxicity [29]. Although definite nephrotoxicity was not observed in the present study, ototoxicity was not evaluated and serum creatinine levels were not repeatedly tested during aminoglycoside therapy in half of the episodes. Therefore, further studies determining the appropriate duration of aminoglycoside therapy, which guarantees both efficacy and safety should be performed. In the ESBL group, rates of susceptibility to oral amoxicillin/clavulanate and trimethoprim/sulfamethoxazole were 66.7 % and 59.1 %, respectively, while 81.8 % (18/22) of ESBL-producing isolates were susceptible to at least one of these two antibiotics. Therefore, the selection of oral antibiotics for UTIs due to ESBL-producing strains should also be further evaluated.

## Conclusions

Childhood UTIs caused by ESBL-producing *E. coli* or *K. pneumoniae* exhibited no significant difference in the clinical and microbiological responses to non-carbapenem antibiotic therapy compared with UTIs caused by non-ESBL producing-strains. Empirical aminoglycoside therapy may be responsible for these results. Aminoglycosides can be an alternative to carbapenems in UTIs caused by ESBL-producing strains; however, further studies regarding oral antibiotic selection as well as the appropriate duration of intravenous aminoglycoside therapy are needed.

## Abbreviations

UTI: Urinary tract infection
ESBL: Extended-spectrum β-lactamase
APN: Acute pyelonephritis
DMSA: ^99m^technetium dimercaptosuccinic acid
VUR: Vesicoureteral reflux
VCUG: Voiding cystourethrography
IQR: Interquartile range
BL: β-lactam
BLI: β-lactamase inhibitor
ID: Infectious disease
